# A High-Performance Spectrometer with Two Spectral Channels Sharing the Same BSI-CMOS Detector

**DOI:** 10.1038/s41598-018-31124-y

**Published:** 2018-08-23

**Authors:** Kai-Yan Zang, Yuan Yao, Er-Tao Hu, An-Qing Jiang, Yu-Xiang Zheng, Song-You Wang, Hai-Bin Zhao, Yue-Mei Yang, Osamu Yoshie, Young-Pak Lee, David W. Lynch, Liang-Yao Chen

**Affiliations:** 1 Department of Optical Science and Engineering, Fudan University, Shanghai, China; 2 Graduate School of IPS, Waseda University, Fukuoka, Japan; 3 Department of Physics, Hanyang University, Seoul, Korea; 4 Department of Physics, Iowa State University, Ames, Iowa USA

## Abstract

Optical spectrometers play an important role in modern scientific research. In this work, we present a two-channel spectrometer with a pixel resolution of better than 0.1 nm/pixel in the wavelength range of 200 to 950 nm and an acquisition speed of approximately 25 spectra per second. The spectrometer reaches a high k factor which characterizes the spectral performance of the spectrometer as k = (working wavelength region)/(pixel resolution) = 7500. Instead of using mechanical moving parts in traditional designs, the spectrometer consists of 8 integrated sub-gratings for diffracting and imaging two sets of 4-folded spectra on the upper and lower parts, respectively, of the focal plane of a two-dimensional backside-illuminated complementary metal-oxide-semiconductor (BSI-CMOS) array detector, which shows a high peak quantum efficiency of approximately 90% at 400 nm. In addition to the advantage of being cost-effective, the compact design of the spectrometer makes it advantageous for applications in which it is desirable to use the same two-dimensional array detector to simultaneously measure multiple spectra under precisely the same working conditions to reduce environmental effects. The performance of the finished spectrometer is tested and confirmed with an Hg-Ar lamp.

## Introduction

The optical spectrometer is a key device in modern scientific research and industrial applications^1–4^. This device has been widely used to extract rich physical information from all types of materials and targets. And it can also be integrated into other types of optical instruments to acquire useful spectral information in a wide variety of fields^5,6^. To further broaden its applications, progress has been made in both precision and miniaturization in the newly developed spectrometers^7–11^.

Generally, spectrometers can be classified according to their underlying operation principles into categories such as grating-based, prism-based, Fabry-Pérot-filter-based, and Fourier-transform-based spectrometers. Being easy to be incorporated into the design of different constructions, gratings are widely used as the key dispersive components in optical spectrometers to achieve high diffraction efficiency and resolution. However, in such systems, to obtain full coverage of the spectral lines in the desired wavelength range with guaranteed resolution in practice, a mechanical motion process with a few scanning steps is still required due to the limitations imposed by the dispersive nature of the optical elements in a traditional spectrometer.

As an alternative approach, great progress has been achieved in recent years using the advanced two-dimensional Si-based charge-coupled-device (CCD) array detectors with an array of sub-gratings instead of the mechanical moving parts that are commonly required to rotate the dispersive optical elements, such as gratings or prisms. By constructing a single integrated grating with multiple sub-gratings, in which the diffraction wavelength λ is blazed at a specially designed angle for each sub-grating, the performance of grating-based spectrometers can be significantly improved through the use of multiply folded spectra to achieve a high and uniform diffraction efficiency over a broad spectral range. In such situation, as it is necessary to measure the whole spectral distribution imaged on the focal plane of the detector, array detectors show considerable advantages over photomultiplier tube (PMT) detectors or other methods that use single photon detector to scan the spectral lines in a measurement process based on the wavelength-scanning interval. Array detectors such as CCD and complementary metal-oxide-semiconductor (CMOS) detectors allow many spectral lines to be recorded simultaneously and optical signals from each pixel to be read out in parallel data acquisition mode^12^. Thus, by exploiting a two-dimensional array detector with a set of sub-gratings to obtain densely folded or dispersed spectral lines, three key instrument properties, namely, (1) a wide working wavelength range, (2) a fine pixel resolution, and (3) a high data acquisition and analysis speed, can be effectively achieved in a single spectrometer without any mechanical moving elements^13,14^.

However, it is critical to mention that in some situations, spectrometers and spectrometer-based instruments need to measure more than one light signals at the same time, for example, to measure the intrinsic optical properties such as the reflectance R, the transmittance T, and the absorption A. According to classical optical principles, the reflectance R is defined as R = I_r_/I_in_, where I_r_ and I_in_ are the intensities of the reflected and incident photon signals, respectively. Usually, I_r_ and I_in_ must be measured independently, either by using a single detector to measure in different time intervals, or using different detectors. However, both methods have their flaws. Measurement in different time intervals will suffer from the time-related errors or errors induced by changing light paths. And it is hard to use different detectors to realize the same quantum efficiency with different environmental conditions. A similar problem exists in applications with requirement of high precision such as the difference frequency generation (DFG). In DFG, the frequency of the output signal significantly relies on the difference between two input light sources with different wavelengths. Thus the accurate and real-time monitoring of both input light sources is needed but often hard to maintain in the same environmental conditions. Consequently, calibration procedures must be performed to correct these errors, which often cannot be easily controlled by users in practice^15^.

Therefore, to overcome these difficulties, in this work, we seek to develop a spectrometer with both more accuracy and improved data acquisition efficiency. To this end, we study a novel compact spectrometer with two spectral channels working in the wavelength region of 200 to 950 nm that is based on an advanced two-dimensional backside-illuminated (BSI−) CMOS array detector, which has better data processing efficiency and lower noise, instead of a CCD array detector. The spectrometer is configured with two optical input channels to enable the imaging of two 4-folded spectra on the same focal plane of the BSI-CMOS array detector. This system is advantageous for the simultaneous measurement and analysis of optical spectra from different or highly correlated spectral light sources at high speed without the need of any mechanical moving parts. It achieves a pixel resolution of better than 0.1 nm/pixel over the entire wavelength region, from 200 nm to 950 nm completely covering the visible region, with a data acquisition speed of approximately 25 spectra per second. This use of an advanced two-dimensional BSI-CMOS array detector shared by multiple spectral channels hope to represent a future trend of advanced spectrometer development.

## Experimental Configuration

**Figure 1:**
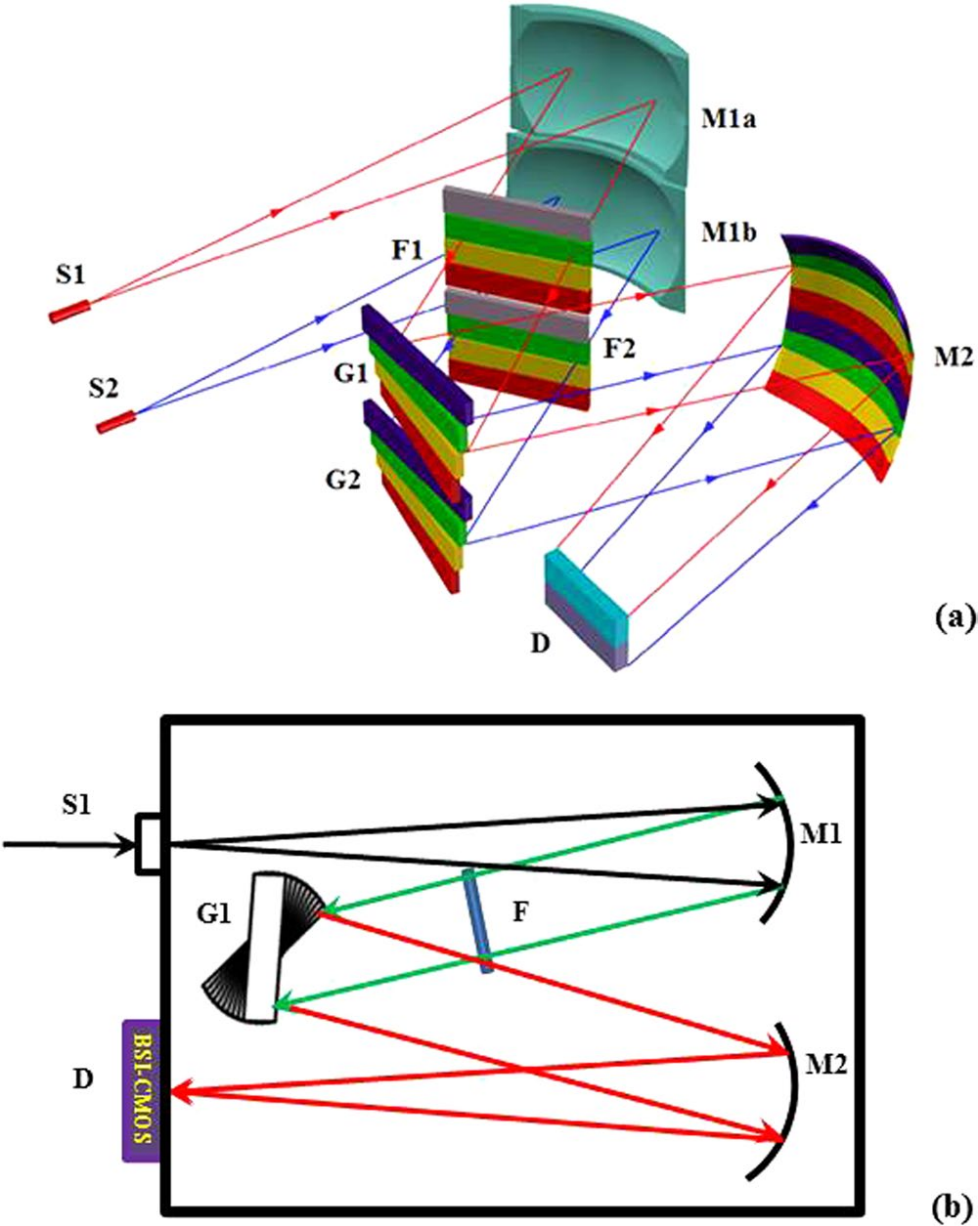
Schematic illustration of the spectrometer system. (**a**) S1 and S2 are two independent optical slits. G1 and G2 are two sets of gratings, each consisting of 4 sub-gratings. The 4-folded spectral lines from G1 and G2 are imaged with high resolution on the upper and lower parts, respectively, of the focal plane of the BSI-CMOS array detector. (**b**) One set of optical elements (S1, G1, mirrors 1 and 2, and filter set F) is arranged such that the spectral lines of channel 1 are imaged on the upper part of the focal plane of the BSI-CMOS detector D. The gray-colored positions shown in F1 and F2 in (a) are blank (without filters).

The specific configuration of the developed spectrometer is shown in Fig. 1. This configuration can be regarded as a combination of multiple sets of Czerny-Turner-type spectrometers integrated in parallel along the vertical direction. The photon-sensing area of the BSI-CMOS array detector is vertically divided into two parts. Two sets of optical paths consisting of different sub-gratings and optical components are present, as shown schematically in Fig. 1(a), which presents a simplified side view of the configuration. The light signals from channels 1 and 2 are input through slits S1 and S2, respectively, which have dimensions of 1.5 mm × 10 μm, and are dispersed by two sets of gratings (G1 and G2), resulting in one set of 4-folded spectral lines being imaged on the upper part of the BSI-CMOS array detector and the other set of spectral lines being imaged on the lower part of the detector. Figure 1(b) shows an overhead view of the light-path configuration using one of the sets as an example. The light emerging from slit 1 is aligned by spherical mirror M1 (with a focal length of 150 mm) such that it is incident on grating set G1 as a set of parallel beams. A set of filters (F), consisting of 3 filters with short cutoff wavelengths of 390 nm, 450 nm, and 650 nm, is placed in front of the 3 lowest gratings to block higher-order diffraction light from entering the gratings. Next, the spectral light dispersed by each grating is focused by toroidal mirror M2, which has two focal lengths of 95 mm and 130 mm in the horizontal and vertical directions, respectively, such that the spectral lines in the 200–950 nm wavelength range are imaged entirely on the upper half of the focusing plane of the CMOS detector. All mechanical parts were designed using computer-aided design (CAD) software and produced with high precision via computer-assisted machining. After assembly and alignment, all parts, including the sub-gratings, were fixed with optical glue to ensure the long-term stability of the system.

**Figure 2:**
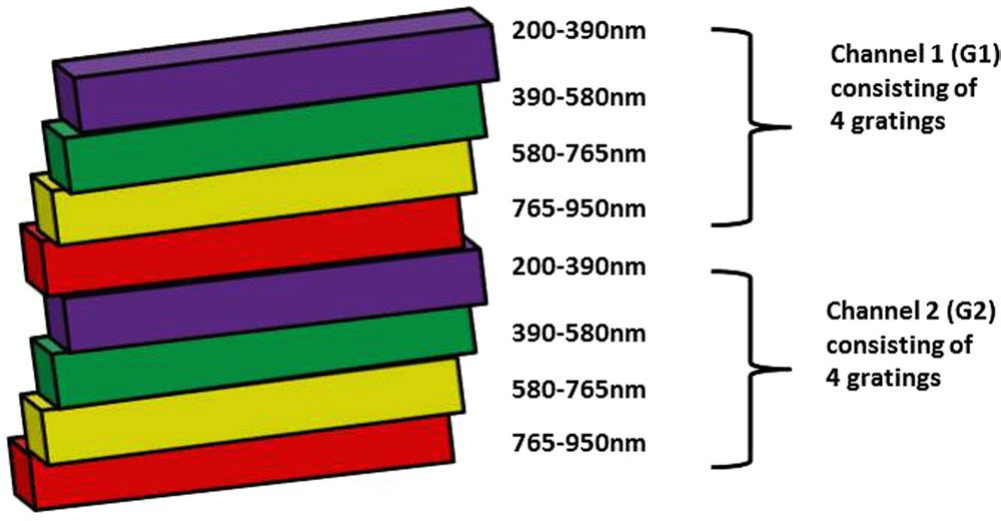
Schematic illustration of the structure of the two sets of gratings. Each of the sets consists of 4 sub-gratings, each corresponding to a different sub-wavelength window.

As shown in Figs 1(a) and 2, each set of gratings consists of 4 sub-gratings (1200 g/mm), each blazed a higher diffraction efficiency at a certain wavelength: 250 nm for the sub-wavelength window of 200–390 nm, 500 nm for the sub-wavelength window of 390–580 nm, and 750 nm for the sub-wavelength windows of 580–765 nm and 765–950 nm. Every sub-grating is fixed at a specially designed angle such that the spectrally dispersed images of the wavelength lines are effectively focused on the corresponding subregions of the image plane of the detector.

In this setup, a Dhyana 90 UV camera^16,17^ is used in combination with a two-dimensional BSI-CMOS array detector with dimensions of 2048 × 2048 pixels and a pixel size of 11 × 11 μm^2^ operating in the 190–1000 nm wavelength region. The detector is set to have a speed of 25 spectra per second, which is the major limitation to the speed of the whole spectrometer. And this detector has a high quantum efficiency, especially in the 200–400 nm wavelength region, with a peak efficiency of approximately 90% at 400 nm, and very low noise. To make full use of its two-dimensional photon-sensing ability, the image plane of the array detector is vertically divided into eight rectangular sub-wavelength windows: the set of four windows in the upper part of the image plane detects the light signals coming from slit 1, and the lower set of windows detects the light from slit 2. Each sub-wavelength window consists of 256 lines of pixels that can be used to measure the spectral light signals. With appropriate analysis, the noise level can be reduced, and optical aberrations can be eliminated.

Theoretical calculations are carried out to determine the resolution of the design. The characteristic linear dispersion relation for a grating system with respect to the wavelength of λ is expressed as

1 $$\frac{{\rm{d}}l}{d\lambda }=\frac{mf}{d\,\cos \,\theta },$$

where *m* (*m* = 1) is the diffraction order of the gratings, *f* (*f* = 95 mm) is the focal length (M2) along the spectrally dispersed direction, *d* is the grating constant (*d* = 1/1200 mm), and *θ* is the diffraction angle. Although the dispersion relation varies slightly with the diffraction angle, the difference between spectral measurements at two wavelengths corresponding to two neighboring pixels can, in principle, be resolved with a maximum resolution of

2 $${\rm{\Delta }}{\lambda }_{\max }=\frac{dw}{f} > \frac{dw\,\cos \,\theta }{f},$$

where *w* is the width of a single pixel, which, in this case, is equal to 11 μm and matches the slit width of 10 μm. The calculated Δλ_max_ is approximately 0.096 nm, implying that the spectrometer has a theoretical resolution of better than 0.096 nm/pixel throughout the entire wavelength region. And in the regions away from the blazed wavelengths, the expected resolution will be slightly lower and does not significantly depend on the wavelength in the whole spectral region. Besides, the resolution should be within the spectral resolving power R of the gratings, given by *R* = λ/Δλ_r_ = *N*, where *N* is the total number of grooves on the grating. The minimum wavelength resolutions Δλ_r_ for *N* = 16800 (grating width = 14 mm) at wavelengths of 200 nm and 950 nm are calculated to be 0.012 nm and 0.057 nm, respectively. Thus, the designed pixel resolution (Δλ_max_ > Δλ_r_) is totally realizable. For each channel, there are 8192 pixels in one dimension, separated into 4 sub-spectral windows, to provide sufficient coverage for the entire spectral range through the pixel-to-wavelength calibration procedure. In accordance with the theoretical expectation, the resolution of better than 0.1 nm/pixel results in a high k factor characterizing the spectral performance of the spectrometer; specifically, k = (working wavelength region)/(pixel resolution) = 7500.

The aberrations due to imperfections in the optical elements and misalignments in the system configuration (such as off-axis aberrations of mirrors and gratings) may not be well addressed in traditional optical designs and can affect the spectral resolution^18,19^. By contrast, in the spectrometer designed in this work, since there are 2048 × 256 pixels available for imaging 256 horizontal spectral lines in each sub-wavelength region, two steps can be applied for correction as follows: First, the standard spectral lines of an elementary light source are used to determine the relationship between the pixel position x(λ_n_) and the wavelength λ_n_. The relationship of line n is then fit in a linear relationship: x(λ_n_) = α_n_ + βλ_n_, where α_n_ is the position constant for line n and β is the dispersion constant of the spectral window. Then, the intensities I_m_(λ_n_) at each pixel position corresponding to the same wavelength λ are summed over the 256 lines to obtain the average intensity I(λ_n_): I(λ_n_) = [∑I_m_(λ_n_)]/256. With this calibration procedure based on the two-dimensional pixel-to-wavelength analysis method, the spectral resolution can be significantly enhanced. Besides, by averaging 256 lines of spectral data, the intensity level of random noise can be lowered by a factor of 16, which is useful in the detection of weak signals.

## Results and Discussion

**Figure 3:**
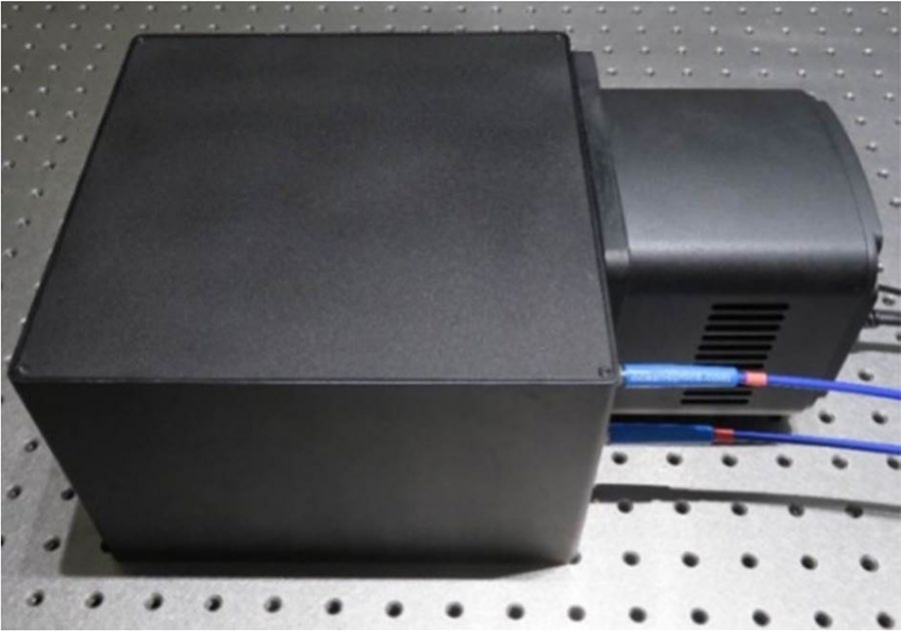
Photograph of the compact spectrometer constructed in accordance with the proposed design.

The compact spectrometer with two spectral channels was successfully constructed in accordance with the proposed design, as shown in Fig. 3. The dimensions are 200 × 200 × 130 mm, not including the camera. Data acquisition software was written and compiled for both calibration and measurement.

**Figure 4:**
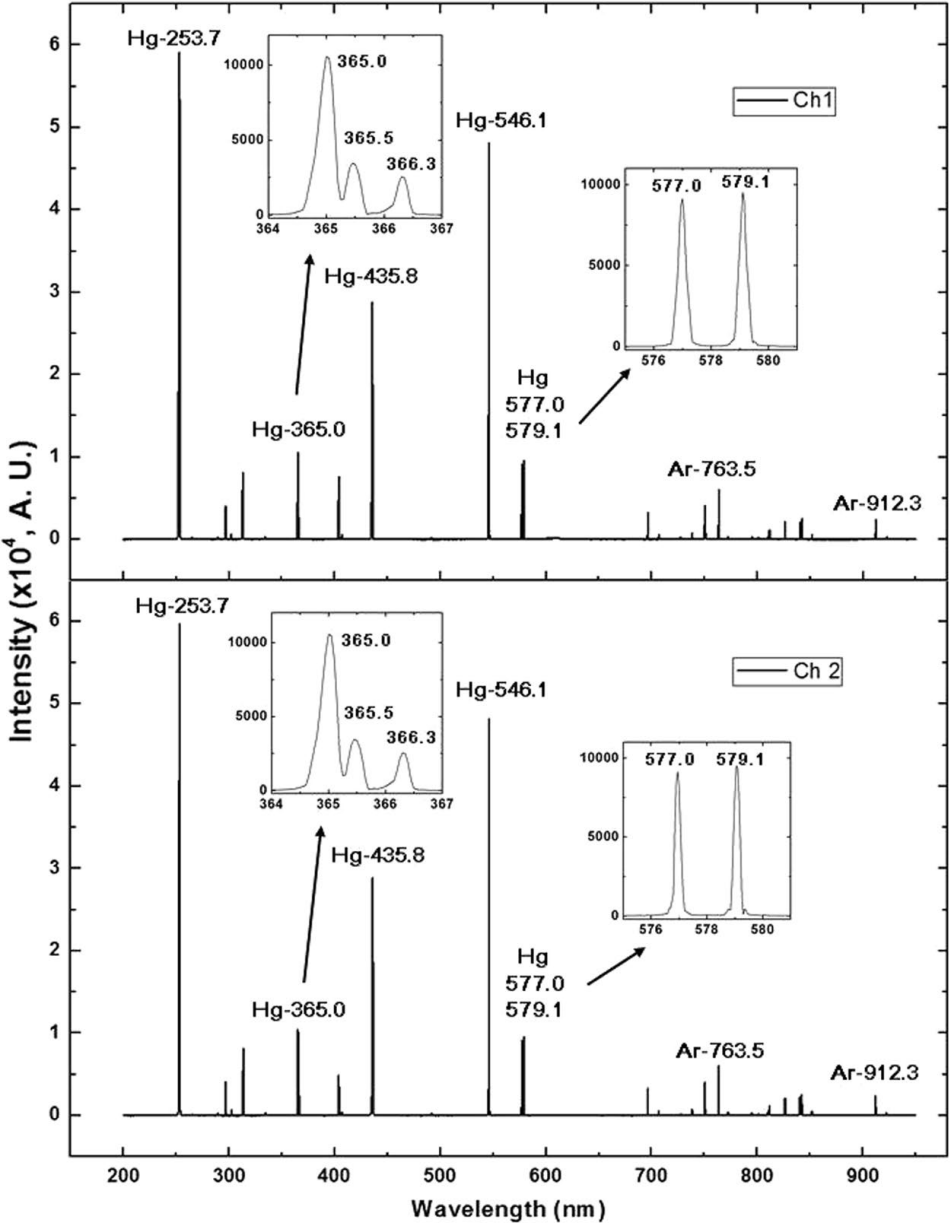
Spectral lines of a Hg-Ar lamp in the wavelength region of 200–950 nm as measured by each channel of the detector, with zoomed views of the well-resolved triple and double spectral lines of the Hg element shown in the insets.

To test the performance of the two-channel optical spectrometer, the spectral lines of a Hg-Ar lamp^20^ in the wavelength region of 200 to 950 nm were simultaneously measured using an optical fiber with a one-to-two configuration, i.e., one end of the fiber was connected to the Hg-Ar light source, and the other two ends of the fiber were connected to the input slits S1 and S2. The spectral results for both channels are shown in Fig. 4, where the insets show zoomed views of the typical triple and double spectral lines of the Hg element. These spectral lines result from the stimulated emission of the Hg atom and are attributed to the transition of the electrons between the internal energy states of the atom. For example, the three spectral lines of Hg located at 365.015 nm, 365.484 nm and 366.328 nm refer to the transitions from the 6*d*^3^*D*_1_, 6*d*^3^*D*_2_ and 6*d*^3^*D*_3_ states to the 6*p*^3^*P*_2_ state, respectively^21^. The measured spectral lines not only can be used to identify the atom existing in the nature without any ambiguity, but also may help to test the resolution limit of spectrometers. It is clear to see in Fig. 4 that all those fine double and triple spectral lines are well resolved. Except for the very weak spectral lines in the 200–250 nm and 925–950 nm wavelength regions due to the limited signal-to-noise ratio of the system, the measured data are in good agreement with the expectations for Hg-Ar spectral lines^20^, including the weak lines in the infrared region commonly ignored by low-precision detections, and confirming that the optical system has been designed correctly.

**Figure 5:**
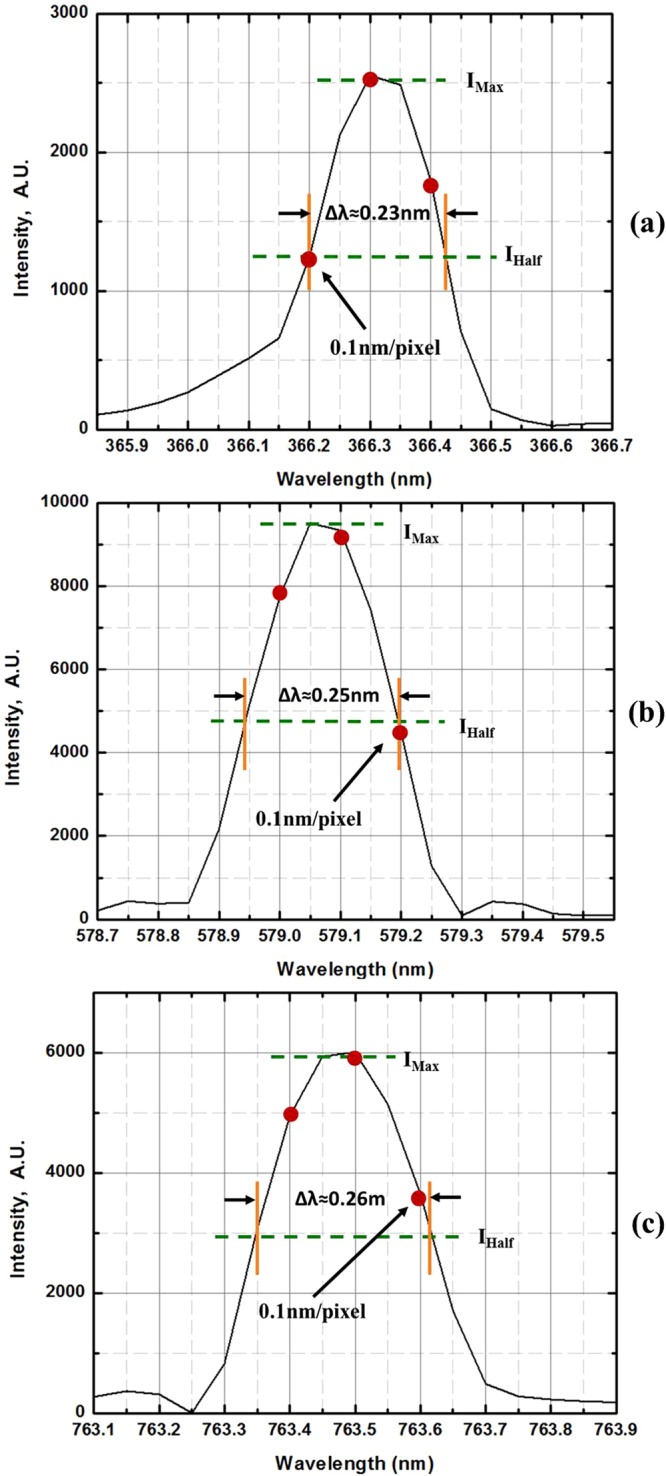
The fine spectral lines located at (**a**) 366.328 nm and (**b**) 579.066 nm for Hg, and at (**c**) 763.511 nm for Ar, respectively, were used to test the pixel and spectral resolutions.

Usually, the measured spectral resolution Δλ is defined by the full width at half maximum (FWHM) of the spectral line and is not equal to the pixel resolution presented in Eq. (2). To avoid the satellite line-broadening effect, which will induce measurement error, the fine 366.3 nm line in the Hg triple-line structure near the 365.0 nm wavelength position, the sharp 579.1 nm line of Hg, and the stronger line of Ar located at 763.5 nm, were used to test the spectral resolution. The FWHM of the spectral line should span at least three pixels (equivalent to three fixed “slits”) with a spectral width of approximately 0.3 nm. In the data analysis, the measured Δλ(FWHM) of 366.3 nm, 579.1 nm and 763.5 nm lines were found to be approximately 0.23 nm, 0.25 nm and 0.26 nm, respectively, as shown in Fig. 5(a–c). This result confirms that the pixel resolution is better than 0.1 nm/pixel [Δλ(FWHM)/3 ≤ Δλ_max_] and indicates that two spectral lines with a wavelength separation of Δλ ≥ 0.3 nm can be clearly resolved, consistent with the measured results for the Hg triple-line structure near the 365.0 nm wavelength position as shown in Fig. 4.

The present design exhibits various advantageous features that are commonly required of an advanced optical spectrometer in practice, i.e., a broad working wavelength range, a high spectral resolution and a real-time measurement capability. Besides, there are more points to mention. First, the spectrometer operates without any mechanical moving parts such as those that are typically present in other spectrometers, which can affect the measurement accuracy due to mechanical errors after long-term usage. Second, an advanced BSI-CMOS array detector of scientific instrument grade with a relatively large size is applied. We take full advantage of the uniformity of the opto-electronic properties of the detector on the imaging plane, with a high quantum efficiency and extremely low noise under a given constant temperature, which can be precisely and uniformly set and controlled down to −25 °C. Third, the optical spectrometer has two independent channels sharing the same detector and operating simultaneously under identical environmental conditions, implying that during data acquisition, the two optical channels will have the same spectral response and thus will require little additional adjustment and correction for time- and feature-dependent errors arising in the measurements due to the use of two different channels for signal detection and processing.

The new spectrometer design proposed in this work not only achieves a high resolution over a broad spectral region with more input channels, but is also compacter, enabling a wider range of applications. In addition, this spectrometer reaches a k factor as high as around 7500 in both channels, proving that the new design has a full utilization of the area for detecting. Moreover, due to the great quantity of high-quantum-efficiency imaging pixels in modern two-dimensional BSI-CMOS array detectors, by decreasing the slit size and configuring more sets of sub-gratings, it is possible for the method presented in this work to be extended to advanced optical spectrometers in which more spectral channels share the same imaging detector with a compact system design, thereby fulfilling the requirements for a broader range of instrumentation applications in many fields of scientific research and industry.

For a two-channel spectrometer, the cost of the mechanical and optical parts and their assembly and alignment will be greater than that for an optical system with only one spectral channel. However, the most expensive part in such an advanced spectrometer is not any of the mechanical or optical elements; instead, it is the scientific-grade two-dimensional array camera with a deep-cooling system. Therefore, for an application in which two spectra are to be measured simultaneously, the total cost of a spectrometer with two spectral channels sharing one two-dimensional array camera with an approximate average power consumption of a few hundred watts will be much lower than the cost of two spectrometers with one camera for each of them to measure the spectra separately.

## Conclusion

In this paper, s new two-channel compact spectrometer with an advanced optical design, in which 8 sub-gratings are integrated with a two-dimensional BSI-CMOS array detector, has been designed and constructed for operation in the wavelength region of 200 to 950 nm. This spectrometer has two optical input channels to enable the simultaneous side-by-side imaging of both 4-folded spectra on the focal plane of the BSI-CMOS array detector. The system has the advantage of enabling the simultaneous measurement and analysis of optical spectra from two different light sources at high speed and without any mechanical moving parts. After calibration, the spectrometer achieves a pixel resolution of better than 0.1 nm/pixel with a data acquisition speed of approximately 25 spectra per second, resulting in a high k factor characterizing its spectral performance (k = (working wavelength region)/(pixel resolution) = 7500), which demonstrates the advantage and potential of this spectrometer for future practical applications in many fields where the high-speed measurement and analysis of multiple spectra are required. The method presented in this work can stimulate research and can be effectively extended to advanced optical spectrometer designs in which more spectral channels share the same imaging detector within a compact system to satisfy the requirements of a broad range of instrumentation applications in many fields of scientific research and industry.
